# Computing distribution of scale independent motifs in biological sequences

**DOI:** 10.1186/1748-7188-1-18

**Published:** 2006-10-18

**Authors:** Jonas S Almeida, Susana Vinga

**Affiliations:** 1 Dept Biostatistics and Applied Mathematics, Univ. Texas MDAnderson Cancer Center, 1515 Holcombe Blvd, Houston TX 77030-4009, USA; 2 Instituto de Engenharia de Sistemas e Computadores: Investigação e Desenvolvimento (INESC-ID), R. Alves Redol 9, 1000-029 Lisboa, Portugal; 3 Departamento de Bioestatística e Informática, Faculdade de Ciências Médicas – Universidade Nova de Lisboa (FCM/UNL), Campo dos Mártires da Pátria 130, 1169-056 Lisboa, Portugal

## Abstract

The use of Chaos Game Representation (CGR) or its generalization, Universal Sequence Maps (USM), to describe the distribution of biological sequences has been found objectionable because of the fractal structure of that coordinate system. Consequently, the investigation of distribution of symbolic motifs at multiple scales is hampered by an inexact association between distance and sequence dissimilarity. A solution to this problem could unleash the use of iterative maps as phase-state representation of sequences where its statistical properties can be conveniently investigated. In this study a family of kernel density functions is described that accommodates the fractal nature of iterative function representations of symbolic sequences and, consequently, enables the exact investigation of sequence motifs of arbitrary lengths in that scale-independent representation. Furthermore, the proposed kernel density includes both Markovian succession and currently used alignment-free sequence dissimilarity metrics as special solutions. Therefore, the fractal kernel described is in fact a generalization that provides a common framework for a diverse suite of sequence analysis techniques.

## Background

The use of iterative functions for scale independent representation of biological sequences was first proposed well over a decade ago [1]. Despite its earlier popularity, that original proposition, designated as Chaos Game Representation (CGR), was soon found objectionable on the grounds of equivalence to standard Markov transition tables [2]. We have subsequently examined that equivalence and have shown that, quite the contrary, it is the Markovian transition that is a special solution of the CGR procedure [3]. The reader is referred to that report for a brief revision of earlier work on iterative functions for representation of sequence succession. The equivalence between iterative maps and genomic signatures (more exactly that the latter comes as a special solution of the former) has also been noted its simpler, and faster implementation [4-7], and it has even lead to a number of web-based and stand alone applications, including a function, CHAOS, available in the popular bioinformatics library EMBOSS [8].

### Why CGR?

Approaching sequence analysis by analyzing the distribution of succession patterns, which is to say, of *L*-tuple (oligomer) frequencies [9], is advantageous when the sequence similarity is low because alignment algorithms cease to recognize common motifs that are inexactly conserved, as recently illustrated for the SCOP protein database [10]. Furthermore, oligomeric frequencies are a natural genomic signature for analysis of collections of isolates [11-13] where, again, the advantages of the CGR representation did not go unnoticed [13]. These observations argue for the value of having a neutral format, one that is scale and succession-independent, to represent Biological sequences. We have used CGR as the starting point to develop just such a general procedure, which we designated as Universal Sequence Maps, USM [14]. The USM procedure provides a bijective mapping (see also [3]) between any symbolic sequence and a unique position in the USM unit hypercube. Furthermore, the distances between map positions were found to be associated with sequence dissimilarity. Because the procedure itself is not dependent on the scale targeted by its analysis (length of motifs, Markov order or memory length, depending on the technique chosen) this is of both fundamental and practical relevance.

### Similarity overestimation

**Figure 1:**
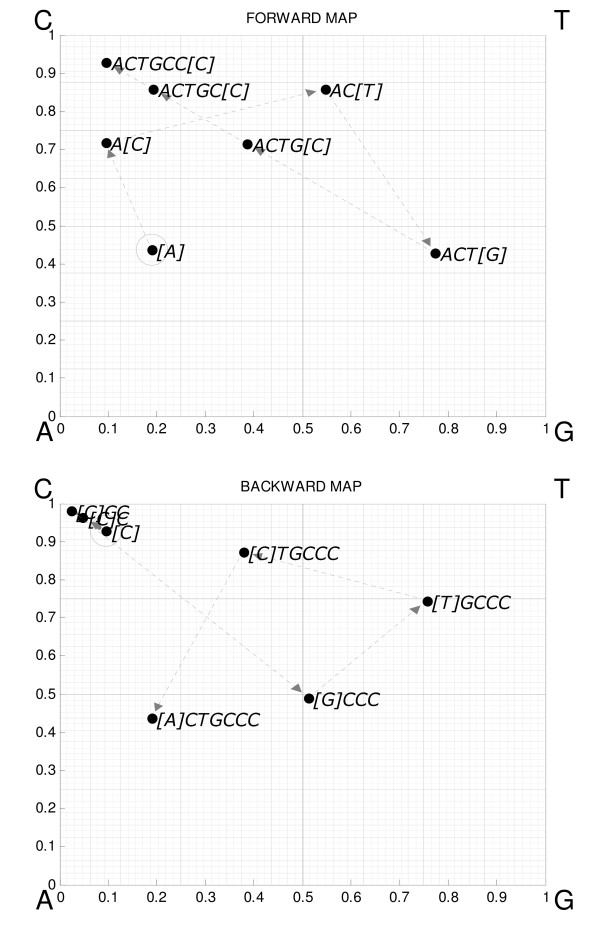
Illustration of the unidirectional USM procedure for the sequence, "ACTGCCC". For a nucleotide sequence, it consists of two iterative CGR operations in each direction, forward and reverse. The circled symbol indicates the first position iterated – see text for discussion on determination of seeding position. Each subsequent position is calculated moving half the distance to the edge with the corresponding unit. As shown in [3] the density of points in the unitary square is a generalization of Markov transition matrices.

The CGR/USM representation of sequences offers fundamental advantages, related with its scale-independency, that make it particularly suitable to investigate the entropy distributions in nucleotide sequences [15]. That study in particular played a significant role in motivating the density kernel development reported here. It was then observed that using symmetric kernels in the Parzen window method, such as the Gaussian distribution function, to represent density of sequence patterns in iterative maps would be affected by some loss of resolution caused by overlap of memory lengths, e.g. different lengths of the sequence pattern being given the same weight because they were at the same Euclidean distance to an arbitrary position in the map. The artifactual loss of resolution can be graphically understood by noting that the projections of two sequence units can be very close to each other in the sequence map for two reasons, only one of them being directly proportional to sequence similarity described in Figure 1. The other, confounding, possibility is that place two units of distinct sequences are placed at close quarters in the sequence map because they happen to be at opposite ends of adjacent quadrants. This rare but unavoidable occurrence causes a bias in previously proposed distance metrics, including our own [3].

The distribution bias caused by the edge effects can be addressed in two different routes. On the one hand it can be modeled and discounted in the final results, as we have done in previous work [14]. Specifically, see Figure 3 of that report for a representation of the (biased) null distribution obtained for different sized alphabets. The alternative solution, which we have also pursued [6] is to identify a Boolean implementation of Universal Sequence Maps, designated as bUSM, which removes the source of distance overestimation at each of the of the scales accommodated by the numerical resolution of the computing environment being used. That report also offers a detailed algebraic description of the causes for the similarity over-estimation for metrics based maximum distances at any dimension (derived from equation 6 in [3]). Neither of those two solutions described, however, helps representing the density distribution of individual sequences such that the sequences themselves can be compared without having to return to the pair-wise distances between their units. The fundamental attraction of such a solution, which we only partially succeeded in [15] using Gaussian Parzen kernels, would be that it captures the fundamental characteristics of the sequence, such as its information content.

### Towards an accurate kernel density function

As shown in previous work discussed above [3,9,14-16], the fact that similar sequence distance is not equidistant (Euclidean) to the preceding position is a serious limitation to sequence comparison. On the other hand, it was also shown [17] that pursuing discriminant analysis using representations that are not constrained by predefined scales or succession orders, even when those scales are systematically screened such as in variable length Markov models [18-20], leads to more accurate models of sequences. The two results put together point to the need for a density kernel that resolves scale (succession order) such that predictive patterns can be investigated more efficiently in the iterative map representation.

In spite of the attractiveness of iterative functions in general, and the bidirectional USM implementation [14] in particular, for enabling the scale independent representation of motifs in biological sequences, its segmentation is still typically approached by considering quadrants that only correspond to Markovian transition. This usage indeed has no fundamental advantage over the better established use of fixed order transition matrices [2]. To go beyond that, the fractal nature [21] set by the consecutive scales that can be spanned by multi-order or fractal order segmentations [3,17] has to be accommodated by the density estimation procedure. As mentioned earlier, we have subsequently approached the investigation of the distributions of motifs of variable length using continuous kernels on the USM positions, such as the Gaussian kernel [15] with only partial success. The limitation of that approach, clearer in the investigation of local entropy, reflected the indetermination of sequence similarity between equidistant positions in the map, which had actually been anticipated, and mathematically modeled, by the original USM proposition [14]. In this report we solve the problem by identifying a kernel for density distribution in the USM space that matches the fractal succession of Markov transition orders. For ease of representation, the procedure will be illustrated for nucleotide sequences, which is also the scale for which unidirectional USM is equivalent to the CGR procedure. This achievement enables the computation of scale independent distribution of motifs in biological sequences which allows different scales to be combined in the same representation of density of motifs in the sequence. The critical advance is that it is no longer affected by the sequence composition itself or, which is the same thing, by the position in the iterative map.

## Methods

### Algorithms and libraries

All algorithms and figures were implemented with original code developed using the programming environment MATLAB 7, Mathworks Inc. The resulting toolbox is and is made freely available at the GeneChaos resource [22] with no restrictions to use or modification. To assist in understanding the proposed algorithms, a function was included that produces the figures presented in this manuscript, e.g. *paper_fig(1) *will produce Figure 1, etc. This function therefore also serves as a tutorial to the usage and interplay of the remaining functions.

### Terminology

This report, and the iterative mapping field in general, mixes terminology from two distinct approaches to sequence analysis which are noteworthy elaboration for the sake of clarity. "Scale" and "resolution" are used as generic terms for a concept that is sometimes precised as "sequence length" or Markovian "order". "Length" is the term used in word-statistics and corresponds to the length of the L-tuple. "Order" describes the same concept but is more commonly used in the context of Markov models. To add to the confusion, L-tuple/word "length" is one unit smaller than "order". For example a simple 4 × 4 transition matrix between nucleotides resolves Markovian succession with order 1 and the conditional probabilities in each of the 16 squares correspond to the frequency of all possible dinucleotides (length 2). Another example, the "scale of L-tuple distribution" designates the length of the tuple for which all frequencies where determined. A variation on this theme is the use of "alphabet size" to access scale: it designates the number of unique symbols available for use in by the sequence. Along the same line of thought, "vocabulary" (not used in this report) would designate the number of possible L-tuples of a given length.

At the origin of this terminology confusion is the fact that both terms, "order" and "length", are originally defined in the context of integer sequence resolution. However the CGR/USM techniques are not restricted to integer resolutions also allow for fractal order/length. Therefore the more generic use of "scale" and "resolution" to overcome the integer presumption. The generalization of scale achieved by iterative maps of discrete sequences was object of some discussion in the early 90's, for example contrast [1] with [2], a topic revised and discussed in [3,9].

#### List of symbols

*u*^*f *^: coordinate in the forward iterative map. The dimension and sequence unit represented are indicated by sub and supra-indexes, ujf(i)
 MathType@MTEF@5@5@+=feaafiart1ev1aaatCvAUfKttLearuWrP9MDH5MBPbIqV92AaeXatLxBI9gBaebbnrfifHhDYfgasaacH8akY=wiFfYdH8Gipec8Eeeu0xXdbba9frFj0=OqFfea0dXdd9vqai=hGuQ8kuc9pgc9s8qqaq=dirpe0xb9q8qiLsFr0=vr0=vr0dc8meaabaqaciaacaGaaeqabaqabeGadaaakeaacqWG1bqDdaqhaaWcbaGaemOAaOgabaGaemOzayMaeiikaGIaemyAaKMaeiykaKcaaaaa@340B@ represents the position in the *j*^*th *^dimension of the iterative map for the *i*^*th *^unit of the sequence.

*u*^*b *^: same as *u*^*b *^but for iterative coordinates in the backward map, that is, obtained by iterating from the end to the beginning of the sequence. For either map, the coordinates fall within the *[0,1] *interval.

Uj(i)
 MathType@MTEF@5@5@+=feaafiart1ev1aaatCvAUfKttLearuWrP9MDH5MBPbIqV92AaeXatLxBI9gBaebbnrfifHhDYfgasaacH8akY=wiFfYdH8Gipec8Eeeu0xXdbba9frFj0=OqFfea0dXdd9vqai=hGuQ8kuc9pgc9s8qqaq=dirpe0xb9q8qiLsFr0=vr0=vr0dc8meaabaqaciaacaGaaeqabaqabeGadaaakeaacqWGvbqvdaqhaaWcbaGaemOAaOgabaGaeiikaGIaemyAaKMaeiykaKcaaaaa@3276@: value of the *j*^*th *^binary digit assigned to the *i*^*th *^unit of the sequence. This positions each unit of the alphabet at an edge of a unitary hypercube [14].

*D *: number of dimensions of each unidirectional map.

N : length of the sequence being represented.

*K *: density kernel, *K(u) *indicates the height of the density distribution in map coordinate *u*.

L : memory length resolution, that is, the length of the segments being resolved. It is equivalent to Markovian order added one unit.

S : kernel smoothing parameter, see equation 3 for definition. The value of S varie between 0, for uniform density, and +∞, where the density distribution is exactly equivalent to a Markov transition table.

## Results

First, the techniques Chaos Game Representation, CGR [1,3], and its bidirectional generalization by Universal Sequence Map, USM [14], will be revisited and illustrated for a small nucleotide sequence. That original report is referenced for the detailed rationale regarding the critical advantage of the bidirectional implementation over the preceding unidirectional solution: all units of a common pattern between two sequences are observed to be equidistant regardless of the individual positions within the sequence. In addition, the USM procedure, more exactly its initialization, will be slightly adjusted to represent motifs in a fashion that is independent of the length of neighboring sequences. Secondly, the discrete density kernel proposed will be described and illustrated with same collections of promoter regions of *Bacillus subtilis *used in the motivating entropy study [15].

### Illustrating iterative map positioning

Universal Sequence Mapping is an iterative procedure that populates a unitary hypercube bijectively: each sequence corresponds to a position in the map, and each map position corresponds to a unique sequence. For nucleotide sequences the hypercube has log_2_(4) = 2 dimensions, that is, it is a unit square. For that case, the original USM procedure in each direction is exactly equal to CGR. The same exercise for a sequence of aminoacids would produce a hypercube with 5 dimensions [14], which is the upper integer of log_2_(20). The edges of the hypercube correspond to the units of the alphabet that compose the sequence and the position is found by moving half the distance between the previous position and the edge corresponding to the unit at the position in the sequence being considered. This procedure, which was formally detailed in a previous report [3], is illustrated in Figure 1 for the sequence ACTGCC. The full USM procedure implements two such mappings, one in the forward and the other in the reverse directions [14].

### Seeding the iterative USM function

The iterative USM procedure described graphically in the previous section and in Figure 1 is formally defined by Equation 1 for an arbitrary sequence of *N *units built from an alphabet with *M *possible symbols.

{ujf(0)=ujb(2)ujf(i)=ujf(i−1)+12(Uj(i)−ujf(i−1))=12ujf(i−1)+12Uj(i)ujb(N+1)=ujf(N−1)ujb(i)=12ujb(i+1)+12Uj(i)Uj(i)∈{0,1}i={1,2,...,N}j={1,2,...,D}     Equation 1
 MathType@MTEF@5@5@+=feaafiart1ev1aaatCvAUfKttLearuWrP9MDH5MBPbIqV92AaeXatLxBI9gBaebbnrfifHhDYfgasaacH8akY=wiFfYdH8Gipec8Eeeu0xXdbba9frFj0=OqFfea0dXdd9vqai=hGuQ8kuc9pgc9s8qqaq=dirpe0xb9q8qiLsFr0=vr0=vr0dc8meaabaqaciaacaGaaeqabaqabeGadaaakeaadaGabaabaeqabaGaemyDau3aa0baaSqaaiabdQgaQbqaaiabdAgaMjabcIcaOiabicdaWiabcMcaPaaakiabg2da9iabdwha1naaDaaaleaacqWGQbGAaeaacqWGIbGycqGGOaakcqaIYaGmcqGGPaqkaaaakeaacqWG1bqDdaqhaaWcbaGaemOAaOgabaGaemOzayMaeiikaGIaemyAaKMaeiykaKcaaOGaeyypa0JaemyDau3aa0baaSqaaiabdQgaQbqaaiabdAgaMjabcIcaOiabdMgaPjabgkHiTiabigdaXiabcMcaPaaakiabgUcaRmaalaaabaGaeGymaedabaGaeGOmaidaamaabmaabaGaemyvau1aa0baaSqaaiabdQgaQbqaaiabcIcaOiabdMgaPjabcMcaPaaakiabgkHiTiabdwha1naaDaaaleaacqWGQbGAaeaacqWGMbGzcqGGOaakcqWGPbqAcqGHsislcqaIXaqmcqGGPaqkaaaakiaawIcacaGLPaaacqGH9aqpdaWcaaqaaiabigdaXaqaaiabikdaYaaacqWG1bqDdaqhaaWcbaGaemOAaOgabaGaemOzayMaeiikaGIaemyAaKMaeyOeI0IaeGymaeJaeiykaKcaaOGaey4kaSYaaSaaaeaacqaIXaqmaeaacqaIYaGmaaGaemyvau1aa0baaSqaaiabdQgaQbqaaiabcIcaOiabdMgaPjabcMcaPaaaaOqaaiabdwha1naaDaaaleaacqWGQbGAaeaacqWGIbGycqGGOaakcqWGobGtcqGHRaWkcqaIXaqmcqGGPaqkaaGccqGH9aqpcqWG1bqDdaqhaaWcbaGaemOAaOgabaGaemOzayMaeiikaGIaemOta4KaeyOeI0IaeGymaeJaeiykaKcaaaGcbaGaemyDau3aa0baaSqaaiabdQgaQbqaaiabdkgaIjabcIcaOiabdMgaPjabcMcaPaaakiabg2da9maalaaabaGaeGymaedabaGaeGOmaidaaiabdwha1naaDaaaleaacqWGQbGAaeaacqWGIbGycqGGOaakcqWGPbqAcqGHRaWkcqaIXaqmcqGGPaqkaaGccqGHRaWkdaWcaaqaaiabigdaXaqaaiabikdaYaaacqWGvbqvdaqhaaWcbaGaemOAaOgabaGaeiikaGIaemyAaKMaeiykaKcaaaGcbaGaemyvau1aa0baaSqaaiabdQgaQbqaaiabcIcaOiabdMgaPjabcMcaPaaakiabgIGiopaacmaabaGaeGimaaJaeiilaWIaeGymaedacaGL7bGaayzFaaaabaGaemyAaKMaeyypa0ZaaiWaaeaacqaIXaqmcqGGSaalcqaIYaGmcqGGSaalcqGGUaGlcqGGUaGlcqGGUaGlcqGGSaalcqWGobGtaiaawUhacaGL9baaaeaacqWGQbGAcqGH9aqpdaGadaqaaiabigdaXiabcYcaSiabikdaYiabcYcaSiabc6caUiabc6caUiabc6caUiabcYcaSiabdseaebGaay5Eaiaaw2haaaaacaGL7baacaWLjaGaaCzcaGqabiab=veafjab=fhaXjab=vha1jab=fgaHjab=rha0jab=LgaPjab=9gaVjab=5gaUjabbccaGiab=fdaXaaa@DAC1@

Each of the unique *M *units of the alphabet are represented by unique binary vector which, graphically, positions them as unique edges of a unitary hypercube with *D *= *log2(M) *dimensions [14]. The reason why the CGR/USM procedure is revisited here is to highlight the novel seeding procedure, by ujb(2)
 MathType@MTEF@5@5@+=feaafiart1ev1aaatCvAUfKttLearuWrP9MDH5MBPbIqV92AaeXatLxBI9gBaebbnrfifHhDYfgasaacH8akY=wiFfYdH8Gipec8Eeeu0xXdbba9frFj0=OqFfea0dXdd9vqai=hGuQ8kuc9pgc9s8qqaq=dirpe0xb9q8qiLsFr0=vr0=vr0dc8meaabaqaciaacaGaaeqabaqabeGadaaakeaacqWG1bqDdaqhaaWcbaGaemOAaOgabaGaemOyaiMaeiikaGIaeGOmaiJaeiykaKcaaaaa@339A@ for the forward iteration and by ujf(N−1)
 MathType@MTEF@5@5@+=feaafiart1ev1aaatCvAUfKttLearuWrP9MDH5MBPbIqV92AaeXatLxBI9gBaebbnrfifHhDYfgasaacH8akY=wiFfYdH8Gipec8Eeeu0xXdbba9frFj0=OqFfea0dXdd9vqai=hGuQ8kuc9pgc9s8qqaq=dirpe0xb9q8qiLsFr0=vr0=vr0dc8meaabaqaciaacaGaaeqabaqabeGadaaakeaacqWG1bqDdaqhaaWcbaGaemOAaOgabaGaemOzayMaeiikaGIaemOta4KaeyOeI0IaeGymaeJaeiykaKcaaaaa@35B2@ for the backward coordinate iteration procedure.

#### Why not seeding at 1/2

In the original CGR proposition [1] the mid coordinate, 1/2, is invariably used as the initial position. Because this position cannot be mapped back to a real sequence this at first appeared as a reasonable proposition even if not fundamentally superior to any of the other boundary positions such as 0 and 1. However, seeding all iterations equally causes an artifactual conservation of the beginning of the sequence which will bias sequence entropy calculations based on map coordinates [15], particularly for small sequences: the first iteration can only produce two coordinates, 1/4 or 3/4, the second iteration will produce one of 4 possibilities: 1/8, 3/8, 5/8 or 7/8, etc. This will cause some extent of artfactual high density at those positions.

#### Other approaches to seeding iterative maps

A possible solution to seed within the domain of possible sequences would be to start with a position randomly collected from a uniform distribution, as indeed used in the original USM paper [14]. However, that too will cause a bias, this time towards missing conservation of initial units in a sequence if that is the case. A negligible few false negatives may be an acceptable outcome for pattern recognition and would have no effect elsewhere in the sequence. However, it falls short of what is required for a kernel generating truly scale independent density distribution of patterns.

#### The solution proposed here

The solution proposed by Equation 1 is to seed the iterative mapping with the reverse coordinates: to seed the first forward coordinate with the next to last backward coordinate for the same dimension and vice versa. Note the first forward coordinate, ujf(1,...)
 MathType@MTEF@5@5@+=feaafiart1ev1aaatCvAUfKttLearuWrP9MDH5MBPbIqV92AaeXatLxBI9gBaebbnrfifHhDYfgasaacH8akY=wiFfYdH8Gipec8Eeeu0xXdbba9frFj0=OqFfea0dXdd9vqai=hGuQ8kuc9pgc9s8qqaq=dirpe0xb9q8qiLsFr0=vr0=vr0dc8meaabaqaciaacaGaaeqabaqabeGadaaakeaacqWG1bqDdaqhaaWcbaGaemOAaOgabaGaemOzayMaeiikaGIaeGymaeJaeiilaWIaeiOla4IaeiOla4IaeiOla4IaeiykaKcaaaaa@372C@, and the last backward coordinate, ujb(...,1)
 MathType@MTEF@5@5@+=feaafiart1ev1aaatCvAUfKttLearuWrP9MDH5MBPbIqV92AaeXatLxBI9gBaebbnrfifHhDYfgasaacH8akY=wiFfYdH8Gipec8Eeeu0xXdbba9frFj0=OqFfea0dXdd9vqai=hGuQ8kuc9pgc9s8qqaq=dirpe0xb9q8qiLsFr0=vr0=vr0dc8meaabaqaciaacaGaaeqabaqabeGadaaakeaacqWG1bqDdaqhaaWcbaGaemOAaOgabaGaemOyaiMaeiikaGIaeiOla4IaeiOla4IaeiOla4IaeiilaWIaeGymaeJaeiykaKcaaaaa@3724@, to be iterated are both the first unit of the sequence, e.g. *i *= 1. Similarly, the last forward coordinate and the first backward coordinate are assigned to the last unit of the sequence, *i *= *N*. Therefore, the new seeding solution can be interpreted as considering that each sequence is preceded and succeeded by its mirror images for the effect of studying local properties. If the sequence is long enough that the numerical resolution of *u*^*f*(*N*) ^is insensitive to the seed value, then the seed value can be determined in practice by simply iterating the last few tens of units of the reverse sequence starting with an arbitrary value. For very short sequences however, Equation 1 has to go through more than one circular iteration, starting from an arbitrary seed value, until the coordinates values converge. This solution causes each unique sequence to have a unique scale independent distribution of patterns where its statistical characteristics can be studied with no need to rebuild the original sequence. This also implies that the coordinates of iterative maps of sequences, as defined by Equation 1, are, fundamentally, steady state solutions. A simple, dramatic, example where this is of consequence is in the positioning of the sequence "A", or "AA" in Figure 1. In the conventional CGR procedure they'd be positioned with coordinates (1/4, 1/4) and (1/8, 1/8) which would place them next to very different, much more heterogeneous, sequences. On the contrary, the solution by seeding as described in Equation 1 will correctly produce the coordinate (0,0). Similarly, a sequence with regular alternation of two units, say "ABABABABAB" should produce well defined density peaks at only two positions, 1/3 and 2/3, which is in fact the steady state solution produced by Equation 1. On the contrary, both CGR and the random seeded USM would produce two trails of values converging to those solutions but not quite reaching them. The fully self-referenced nature of the modified USM construction is also reflected in the observation that the steady state solutions invariably produce ujf(1)=ujb(1)
 MathType@MTEF@5@5@+=feaafiart1ev1aaatCvAUfKttLearuWrP9MDH5MBPbIqV92AaeXatLxBI9gBaebbnrfifHhDYfgasaacH8akY=wiFfYdH8Gipec8Eeeu0xXdbba9frFj0=OqFfea0dXdd9vqai=hGuQ8kuc9pgc9s8qqaq=dirpe0xb9q8qiLsFr0=vr0=vr0dc8meaabaqaciaacaGaaeqabaqabeGadaaakeaacqWG1bqDdaqhaaWcbaGaemOAaOgabaGaemOzayMaeiikaGIaeGymaeJaeiykaKcaaOGaeyypa0JaemyDau3aa0baaSqaaiabdQgaQbqaaiabdkgaIjabcIcaOiabigdaXiabcMcaPaaaaaa@3B9C@ and ujf(N)=ujb(N)
 MathType@MTEF@5@5@+=feaafiart1ev1aaatCvAUfKttLearuWrP9MDH5MBPbIqV92AaeXatLxBI9gBaebbnrfifHhDYfgasaacH8akY=wiFfYdH8Gipec8Eeeu0xXdbba9frFj0=OqFfea0dXdd9vqai=hGuQ8kuc9pgc9s8qqaq=dirpe0xb9q8qiLsFr0=vr0=vr0dc8meaabaqaciaacaGaaeqabaqabeGadaaakeaacqWG1bqDdaqhaaWcbaGaemOAaOgabaGaemOzayMaeiikaGIaemOta4KaeiykaKcaaOGaeyypa0JaemyDau3aa0baaSqaaiabdQgaQbqaaiabdkgaIjabcIcaOiabd6eaojabcMcaPaaaaaa@3C06@. However, exploring the bidirectional density distributions is beyond the scope of this report.

### Construction of density kernel

The shape of the density kernel should match the fractal nature of the iterative USM function itself. The solution reported here will first be described for a USM coordinate, and illustrated for an arbitrary coordinate of the map, say the horizontal dimension of the forward map in Figure 1. The value, *K*, of the proposed Kernel function (Equation 2) in map coordinate position *u*, has two user-defined parameters, memory length, *L*, and smoothing, *S*, which is the ratio between the areas assigned to two consecutive Markov orders (e.g. *S *= *2 *implies the kernel density area assigned to order *i *≤ *L-1 *is twice the area assigned to order *i-1*).

K(u)=∑j=1N∑i=1L{H(i,D,L,S)←LB(i,xj)<u<UB(i,xj)0←otherwise     Equation 2
 MathType@MTEF@5@5@+=feaafiart1ev1aaatCvAUfKttLearuWrP9MDH5MBPbIqV92AaeXatLxBI9gBaebbnrfifHhDYfgasaacH8akY=wiFfYdH8Gipec8Eeeu0xXdbba9frFj0=OqFfea0dXdd9vqai=hGuQ8kuc9pgc9s8qqaq=dirpe0xb9q8qiLsFr0=vr0=vr0dc8meaabaqaciaacaGaaeqabaqabeGadaaakeaacqWGlbWscqGGOaakcqWG1bqDcqGGPaqkcqGH9aqpdaaeWbqaamaaqahabaWaaiqaaqaabeqaaiabdIeaijabcIcaOiabdMgaPjabcYcaSiabdseaejabcYcaSiabdYeamjabcYcaSiabdofatjabcMcaPiabgcziSkabdYeamjabdkeacjabcIcaOiabdMgaPjabcYcaSiabdIha4naaBaaaleaacqWGQbGAaeqaaOGaeiykaKIaeyipaWJaemyDauNaeyipaWJaemyvauLaemOqaiKaeiikaGIaemyAaKMaeiilaWIaemiEaG3aaSbaaSqaaiabdQgaQbqabaGccqGGPaqkaeaacqaIWaamcqGHqgcRcqWGVbWBcqWG0baDcqWGObaAcqWGLbqzcqWGYbGCcqWG3bWDcqWGPbqAcqWGZbWCcqWGLbqzaaGaay5EaaaaleaacqWGPbqAcqGH9aqpcqaIXaqmaeaacqWGmbata0GaeyyeIuoaaSqaaiabdQgaQjabg2da9iabigdaXaqaaiabd6eaobqdcqGHris5aOGaaCzcaiaaxMaaieqacqWFfbqrcqWFXbqCcqWF1bqDcqWFHbqycqWF0baDcqWFPbqAcqWFVbWBcqWFUbGBcqqGGaaicqWFYaGmaaa@7F88@

**Figure 2:**
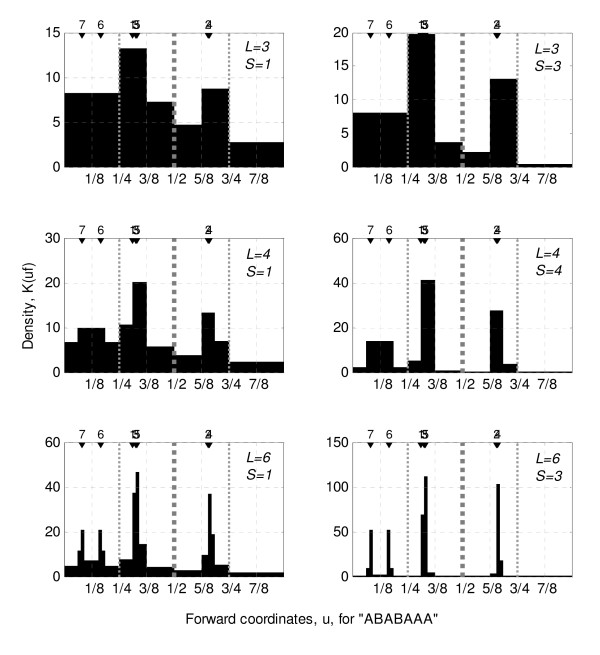
Illustration of the Kernel density, K, for a small binary sequence, "ABABAAA", along its single USM axis, using different values for memory length, *L*, and smoothing, *S*. The same seven coordinates are used in all plots which implies that each of the 6 density plots have a similar area of 7 kernel units.

**Figure 3:**
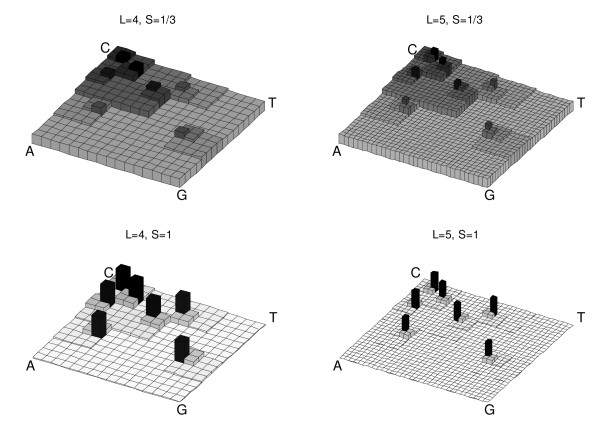
Determination of Kernel density, Equation 2, in the forward map of the sequence "ACTGCCC" used to produce Figure. To illustrate the effect of using different settings for memory length, *L*, and smoothness, *S*, The kernel density was determined for the four different combinations of *L *= {4, 5} and *S *= {1, 1/3}.

The parameter *D *is the number of dimensions of the unitary USM hypercubes (e.g. *d *= *2 *for the example in Figure 1) and the expression in Equation 2 simply states that the kernel density value in position *u *is obtained by adding the values of *H*, for each of the orders up to *L-1*, which makes it a scale dependent height function, for the number of elements of the kernel training dataset, *x*, that are positioned within a scale dependent neighborhood confined by lower and upper boundaries, *LB *and *UB*, respectively. The choice of memory length, *L*, of the kernel, sets the resolution of the density function. This is graphically reflected by the finer grain of the density distribution for higher values of *L *in Figure 2 and Figure 3.

**Figure 4:**
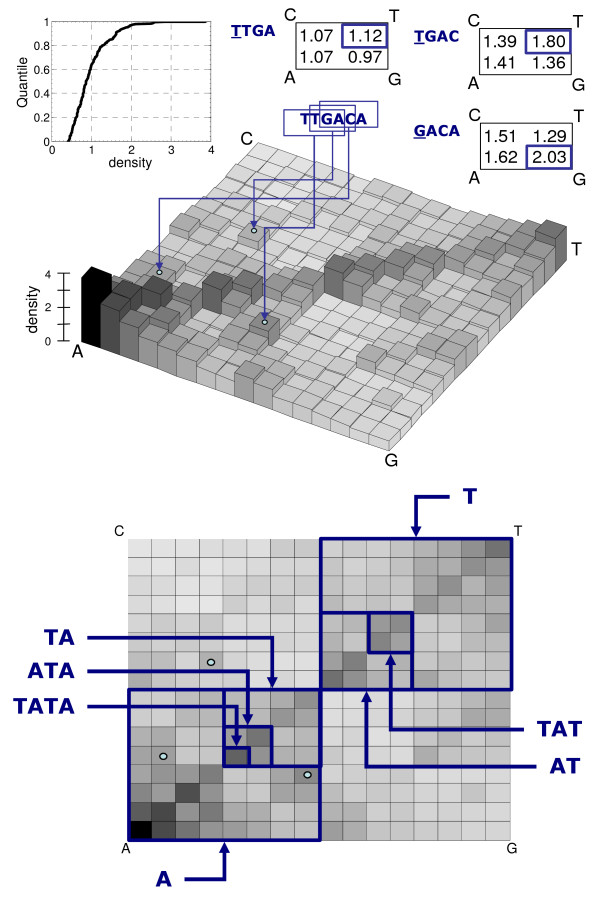
Kernel density for *L *= 4 and *S *= 1 applied to the concatenation of 20 promoter regions of *Bacillus subtilis *(see Discussion). The density is displayed both as a 3D bar (top) and as a 2D gray scale heat map (bottom). The accurate capturing of conserved tetranucleotide segments is illustrated for the TATA-box in the latter view, and for the TTGACA binding site at position -35 in the former. The two views also illustrate the two types of decomposition of conserved sequences. For the TTGACA sequence the decomposition is performed for the resolution of the kernel (L = 4) and all 3 tetranucleotides embedded in the 6 unit sequence are identified. The density scale is normalized to the length of the sequence so the average height is one unit – which is to say that the area of the density distribution is, as it should for a unit square base, unitary by definition. The three tables at the top detail the densities of the possible tetranucleotides for each of the trinucleotide quadrants. It can be observed that in each of them the conserved segment invariably has the highest density. The decomposition of the TATA-box, in the bottom view is instead illustrated for a succession of scales, from mononucleotide to tetranucleotide. The cumulative distribution of densities is displayed at the top left, disclosing a skew towards lower values, with over 60% of densities are below the unit average.

As will be shown next, the kernel volume defined by this surface is equal to the number of points (sequence units), *N*, of the kernel-training dataset *x*. This result, strictly considered, disqualifies *K *as a kernel density function as kernel density volumes are unitary by definition. There are a number of reasons why having a volume that is the number of sequence units is desirable, particularly when sequences of different lengths are being compared. A compliant alternative definition of *K *is in any case obtained by dividing the expression in Equation 2, by the total length of the training sequences, *N*. This alternative will not be explicitly explored here because the scale alteration is so straight forward that it can easily be applied to any of the results reported here. The 2D density plots are offered without a scale in the z-axis to highlight the inconsequence of the correction. On the other hand, when multiple sequences are plotted together, as in Figure 4, the effect is that that the same motif in two sequences is represented with the same density height, Equation 3, even if the two sequences have very different lengths.

The kernel density definition in Equation 2 is completed by two more expressions, Equation 3 and Equation 4, where the height function and its boundaries are detailed. The kernel density height function, Equation 3, establishes the step height added at each memory length smaller or equal to the value of L. It is useful to recall that the memory length is one unit smaller than the Markovian order, e.g. for nucleotide sequences, memory length one corresponds to mono nucleotide frequencies, memory length two corresponds to di-nucleotide frequencies, which populate a first order Markov transition table, and so on.

H(i,D,L,S)=(2D/S)i∑r=0LS−L     Equation 3
 MathType@MTEF@5@5@+=feaafiart1ev1aaatCvAUfKttLearuWrP9MDH5MBPbIqV92AaeXatLxBI9gBaebbnrfifHhDYfgasaacH8akY=wiFfYdH8Gipec8Eeeu0xXdbba9frFj0=OqFfea0dXdd9vqai=hGuQ8kuc9pgc9s8qqaq=dirpe0xb9q8qiLsFr0=vr0=vr0dc8meaabaqaciaacaGaaeqabaqabeGadaaakeaacqWGibascqGGOaakcqWGPbqAcqGGSaalcqWGebarcqGGSaalcqWGmbatcqGGSaalcqWGtbWucqGGPaqkcqGH9aqpdaWcaaqaaiabcIcaOiabikdaYmaaCaaaleqabaGaemiraqeaaOGaei4la8Iaem4uamLaeiykaKYaaWbaaSqabeaacqWGPbqAaaaakeaadaaeWbqaaiabdofatnaaCaaaleqabaGaeyOeI0IaemitaWeaaaqaaiabdkhaYjabg2da9iabicdaWaqaaiabdYeambqdcqGHris5aaaakiaaxMaacaWLjaacbeGae8xrauKae8xCaeNae8xDauNae8xyaeMae8hDaqNae8xAaKMae83Ba8Mae8NBa4MaeeiiaaIae83mamdaaa@575D@

The boundary values set by the functions LB and UB, Equation 4, define the neighborhood of a training sequence unit, that is, neighborhood to its USM position, *x*, which will have the corresponding value of *H*, Equation 3, added to the kernel density height, as detailed in Equation 2.

LB(i,x)=floor(x⋅2i)2iUB(i,x)=floor(x⋅2i)+12i     Equation 4
 MathType@MTEF@5@5@+=feaafiart1ev1aaatCvAUfKttLearuWrP9MDH5MBPbIqV92AaeXatLxBI9gBaebbnrfifHhDYfgasaacH8akY=wiFfYdH8Gipec8Eeeu0xXdbba9frFj0=OqFfea0dXdd9vqai=hGuQ8kuc9pgc9s8qqaq=dirpe0xb9q8qiLsFr0=vr0=vr0dc8meaabaqaciaacaGaaeqabaqabeGadaaakqaabeqaaiabdYeamjabdkeacjabcIcaOiabdMgaPjabcYcaSiabdIha4jabcMcaPiabg2da9maalaaabaGaemOzayMaemiBaWMaem4Ba8Maem4Ba8MaemOCaiNaeiikaGIaemiEaGNaeyyXICTaeGOmaiZaaWbaaSqabeaacqWGPbqAaaGccqGGPaqkaeaacqaIYaGmdaahaaWcbeqaaiabdMgaPbaaaaaakeaacqWGvbqvcqWGcbGqcqGGOaakcqWGPbqAcqGGSaalcqWG4baEcqGGPaqkcqGH9aqpdaWcaaqaaiabdAgaMjabdYgaSjabd+gaVjabd+gaVjabdkhaYjabcIcaOiabdIha4jabgwSixlabikdaYmaaCaaaleqabaGaemyAaKgaaOGaeiykaKIaey4kaSIaeGymaedabaGaeGOmaiZaaWbaaSqabeaacqWGPbqAaaaaaOGaaCzcaiaaxMaaieqacqWFfbqrcqWFXbqCcqWF1bqDcqWFHbqycqWF0baDcqWFPbqAcqWFVbWBcqWFUbGBcqqGGaaicqWF0aanaaaa@707D@

Before illustrating the calculation of the kernel density for multi-dimensional USM hypercube it is useful to illustrate the procedure for the one-dimensional example of a binary sequence such as 'ABABAAA'. The corresponding USM forward coordinates would be [0.3138 0.6569 0.3284 0.6642 0.3321 0.1661 0.0830] and the corresponding kernel density, Equation 2, for all positions in the one-dimensional USM map are shown in Figure 2 for different values of memory length, *L*, and smoothing, *S*.

Figure 2 illustrates how the choice of parameters will set both the resolution and detail of the pattern representation. If smoothing is set to +∞ then the kernel density will be distributed between the different fractions exactly as it would in a Markov transition matrix with the same memory length. This becomes clearer when a two dimension example is used such as the more familiar representation of nucleotide sequences. To illustrate this procedure, Equation 2 was applied to the forward map of a small nucleotide sequence represented in Figure 1, which results in the density distribution represented in Figure 3.

## Discussion

A novel kernel density method to measure oligomeric frequency in a iterative sequence maps of biological sequences (Chaos Game Representation or its generalization to alphabets longer than 4 units, Universal sequence Maps) was described and summarily illustrated. However, the illustration would not be complete without mapping the promoter regions of *Bacillus subtilis *and the recognition of the TATA box in the same sequences used in the preceding report [15], which motivated the development described here. This discussion will therefore focus on the representation and decomposition of sequence conservation, which can be detected by unlikely repetition of the conserved segment of because the conserved segment has an unlikely composition in the context of the remaining sequence. Accordingly, the illustration in Figure 4 uses the same 20, 100 unit long upstream promoter regions of *B.subtilis *obtained from [23,24], all having a known promoter sequence constituted by the sub-string TTGACA-(space)-TATAAT with at most one substitution (known as the TATA-box). The entropic properties of those sequences were discussed in the preceding work [15], were they were designated by the *Es *symbol. For the sake of reference, the *Es *concatenation is embedded in the software library provided with this report, and is retrieved when using the illustrative function *paper_fig(4)*, which reproduces Figure 4 (this function can be used to reproduce the other three figures too, see Methods). The volume under the density distribution is, by definition, unitary (the normalized height is obtained by dividing H, equation 3, by N, the total number of sequence units). Therefore, the average value of the matrix underneath the 3D bar plot in Figure 4 is also unitary and sets the scale for the representation (scaled height axis is represented in the 3D view of the density distribution represented in Figure 4).

Two important issues for pattern recognition in sequences are raised by this illustration and warrant discussion even if they fall outside the strict reporting of a kernel density distribution method. Firstly, it is clear that for any fixed resolution, *L*, all conserved segments of longer length will have its *L*-long sub-segments represented as peaks scattered throughout the distribution. As a consequence, the choice of value for the smooth parameter, *S*, should be set as to maximize the recognition of an objective quantity, such as information content. When scanning different scales, by using various values for *L*, the optimal value of S would also be different, as it would be dependent on the information content encoded at that scale. Secondly, the shorter sub-segments of a conserved segment of length *L*, will set the base height for the quadrants where the conserved *L*-long segment is inserted. Therefore, the availability of a density distribution kernel for the projection of sequences in a continuous space also creates the opportunity to devise de-embedding schemes that will pinpoint the location of conservation for arbitrary target resolutions.

## Conclusion

As in previous methodological developments associated with this technique, the more conventional, Markovian, solutions emerge as special formulations of the proposed novel methodology. For example, using very large smoothing parameters, *S*~ +*∞*, will exactly identify a Markov transition table of order *L-1*. The development of this kernel comes in the sequence of generalizing it beyond non-nucleotide alphabets and then screening different scales to describe its global entropic properties. Each step in this progression came with adjustments or reinterpretations of the original CGR procedure. This one is no exception and a more balanced, fully self-referenced, solution to the seeding of the iterative procedure was found that suggests that CGR/USM coordinates may best be sought as steady state solutions. However, for all but the shortest sequences this is of no computational consequence. Finally, a software library in a user-friendly programming language (Matlab code has a high-level pseudo-language appearance) is disseminated with this report to facilitate both independent use of the scale variant density distributions and further development of the method itself.

## References

[B1] Jeffrey HJ (1990). Chaos game representation of gene structure. Nucleic Acids Res.

[B2] Goldman N (1993). Nucleotide, dinucleotide and trinucleotide frequencies explain patterns observed in chaos game representations of DNA sequences. Nucleic Acids Res.

[B3] Almeida JS, Carrico JA, Maretzek A, Noble PA, Fletcher M (2001). Analysis of genomic sequences by Chaos Game Representation. Bioinformatics.

[B4] Dufraigne C, Fertil B, Lespinats S, Giron A, Deschavanne P (2005). Detection and characterization of horizontal transfers in prokaryotes using genomic signature. Nucleic Acids Res.

[B5] Deschavanne PJ, Giron A, Vilain J, Fagot G, Fertil B (1999). Genomic signature: characterization and classification of species assessed by chaos game representation of sequences. Mol Biol Evol.

[B6] Schwacke J, Almeida JS (2002). Efficient Boolean implementation of universal sequence maps (bUSM). BMC Bioinformatics.

[B7] Hess CM, Gasper J, Hoekstra HE, Hill CE, Edwards SV (2000). MHC class II pseudogene and genomic signature of a 32-kb cosmid in the house finch (Carpodacus mexicanus). Genome Res.

[B8] Rice P, Longden I, Bleasby A (2000). EMBOSS: the European Molecular Biology Open Software Suite. Trends Genet.

[B9] Vinga S, Almeida J (2003). Alignment-free sequence comparison-a review. Bioinformatics.

[B10] Vinga S, Gouveia-Oliveira R, Almeida JS (2004). Comparative evaluation of word composition distances for the recognition of SCOP relationships. Bioinformatics.

[B11] Karlin S, Mrazek J, Campbell AM (1997). Compositional biases of bacterial genomes and evolutionary implications. J Bacteriol.

[B12] Karlin S, Mrazek J, Gentles AJ (2003). Genome comparisons and analysis. Curr Opin Struct Biol.

[B13] Wang Y, Hill K, Singh S, Kari L (2005). The spectrum of genomic signatures: from dinucleotides to chaos game representation. Gene.

[B14] Almeida JS, Vinga S (2002). Universal sequence map (USM) of arbitrary discrete sequences. BMC Bioinformatics.

[B15] Vinga S, Almeida JS (2004). Renyi continuous entropy of DNA sequences. J Theor Biol.

[B16] Vinga S, Gouveia-Oliveira R, Almeida JS (2004). Comparative evaluation of word composition distances for the recognition of SCOP relationships.. Bioinformatics.

[B17] Tino P, Dorffner G (2001). Predicting the Future of Discrete Sequences from Fractal Representations of the Past. Machine Learning.

[B18] Cowell LG, Davila M, Kepler TB, Kelsoe G (2002). Identification and utilization of arbitrary correlations in models of recombination signal sequences. Genome Biol.

[B19] Bejerano G (2004). Algorithms for variable length Markov chain modeling. Bioinformatics.

[B20] Bühlmann P, Wyner AJ (1999). Variable length Markov chains. Annals of Statistics.

[B21] Gutierrez JM, Rodriguez MA, Abramson G (2001). Multifractal analysis of DNA sequences using a novel chaos-game representation. Physica A: Statistical Mechanics and its Applications.

[B22] Almeida JS GeneChaos.ORG resource. http://genechaos.org.

[B23] Helmann JD (1995). Compilation and analysis of Bacillus subtilis sigma A-dependent promoter sequences: evidence for extended contact between RNA polymerase and upstream promoter DNA. Nucleic Acids Res.

[B24] Vanet A, Marsan L, Sagot MF (1999). Promoter sequences and algorithmical methods for identifying them. Res Microbiol.

